# Endovascular Treatment of Spontaneous and Isolated Infrarenal Acute Aortic Syndrome with Unibody Aortic Stent-Grafts

**DOI:** 10.1007/s00268-020-05754-1

**Published:** 2020-09-03

**Authors:** Felice Pecoraro, Ettore Dinoto, Domenico Mirabella, Francesca Ferlito, Arduino Farina, David Pakeliani, Mario Lachat, Francesca Urso, Guido Bajardi

**Affiliations:** 1 Department of Surgical, Oncological and Oral Sciences (Di.Chir.On.S.), University of Palermo, Via L. Giuffrè, 5, 90100 Palermo, Italy; 2 Vascular Surgery Unit, AOUP “P. Giaccone”, Palermo, Italy; 3 Vascular Surgery Unit, Ospedali Riuniti Villa Sofia-Cervello, Palermo, Italy; 4 Aortic Center Hirslanden, Zurich, Switzerland

## Abstract

**Introduction:**

Spontaneous acute aortic syndrome (IAAS) is rarely localized in the infrarenal aorta. The endovascular approach is preferred over conventional open surgery with fewer complications. However, dedicated endovascular devices for IAAS treatment are unavailable. The aim was to report a large single-center experience using unibody stent-grafts to address IAAS.

**Methods:**

From April 2016 to March 2019, a retrospective analysis of patients presenting spontaneous and isolated IAAS was performed. Patients addressed with the unibody stent-graft (AFX endovascular AAA system; Endologix Inc., Irvine, CA) were included in the study. Indications to IAAS treatment were persistent symptoms and/or dilated abdominal aorta (>3 cm). The measured outcomes were technical success; early outcomes (<30 days) including mortality, morbidity, symptoms recurrence, and endoleak occurrence; and late outcomes (>30 days) including mortality, symptoms recurrence, endoleak occurrence, stent-graft patency, and survival. Median follow-up was 23.77 ± 10 months.

**Results:**

Twenty-one patients with IAAS were included. Indications to treatment were symptoms in 14 (67%) patients and dilated abdominal aorta in 7 (33%). Technical success was achieved in all cases. No perioperative mortality and 1 (4.8%) early femoral access complication was encountered. During the follow-up were registered 1 (4.8%) aortic unrelated death and 1 (4.8%) stent-graft limb stenosis. The 36 months estimated survival and freedom from reintervention were 92% (CI: 37–43; SE: 1.7) and 94% (CI: 37–44; SE: 1.7), respectively.

**Conclusions:**

The endovascular treatment of IAAS with unibody stent-graft (AFX endovascular AAA system; Endologix Inc.) is safe and effective with promising mid-term outcomes. The use of unibody stent-grafts expands the endovascular indication, despite the usual anatomic IAAS features. Larger studies with longer follow-up are required to validate the outcomes of the reported technique.

## Introduction

The term “acute aortic syndrome” (AAS) is generally employed to describe life-threatening aortic diseases, that includes: intramural hematoma (IMH), penetrating aortic ulcer (PAU), and aortic dissection (AD) [1]. Despite different pathophysiology of such entities, a causal relationship has been proposed linking these three conditions, which represents a different stage of the same evolutive disease [2]. ADs represent the majority of AAS with a reported incidence of 2.6–3.5 cases per 100,000 person-years [3]. ADs are more frequently localized in the ascending aorta (70%) followed by the descending aorta (20%) and the aortic arch (7%). Isolated abdominal aortic dissection (IAAD) is an extremely rare occurrence with a reported prevalence of 1.1–4% and in 91–96% present a spontaneous nature [4–6]. IMH accounts for approximately 6–10% of all AAS [7]. The true incidence of PAU has yet to be determined. However, abdominal PAU is less frequent than thoracic PAU with an estimated incidence of 1–5% [8].

As a result, the infrarenal localization of AAS is even rarer with limited and varied literature on the topic and unclear indications for medical, surgical, and endovascular treatment.

When an invasive IAAD treatment is indicated, the endovascular approach is privileged with fewer complications and mortality rates in the short and long term [5, 6]. Definitely no clear indication or evidence of superiority exists on the type of endovascular materials to employ.

A single-center experience of isolated infrarenal acute aortic syndrome (IAAS) treatment by endovascular means using unibody stent-graft is reported. The study aims to assess the feasibility of addressing IAAS lesions with a commercially available unibody stent-graft (AFX endovascular AAA system; Endologix Inc., Irvine, CA) and report the mid-term outcomes.

## Materials and methods

From April 2016 to March 2019, data from consecutive patients presenting IAAS were prospectively collected, inserted into standardized piloted forms, and retrospectively analyzed. Patients treated with unibody stent-graft (AFX endovascular AAA system; Endologix Inc.) and presenting IAAS involving the infrarenal aorta were included in the study. AAS was defined according to the International Registry of Acute Aortic Dissection (IRAD) and included aortic dissection (AD), intramural hematoma (IMH), and penetrating aortic ulcer (PAU) [9]. Suprarenal AAS, post-traumatic AAS, or iatrogenic AAS was excluded. Patients managed with conservative therapy and open surgical treatments or addressed by endovascular means without the use of unibody aortic stent-grafts were also excluded from the current study. All patients gave the informed consent for the procedure itself, anonymous data collection, and analysis. According to the internal review board, the retrospective and anonymized nature of the study did not require medical ethical committee approval.

Indications to IAAS treatment were symptomatic IAAS and/or an associated dilated abdominal aorta (>3 cm). Symptomatic IAAS were defined as abdominal/back pain, organ ischemia, distal embolization, and aortic rupture.

Demographic, comorbidities, and clinical data were collected. The preoperative types of imaging studies, procedure details, type of intervention, type of anesthesia, blood transfusions, medical therapy, and length of stay (LOS) were recorded.

Renal function was calculated according to the Chronic Kidney Disease Epidemiology Collaboration (CKD-EPI) formula [10]. Cardiac and respiratory function was assessed using The New York Heart Association (NYHA) heart function [11] and the Global Initiative for Chronic Obstructive Lung Disease (GOLD) [12].

The measured outcomes were technical success, early (within 30 days) and late (after 30 days) outcomes. Technical success was defined as procedure completion as intended with the complete exclusion of the IAAS and without unplanned additional maneuvers. Early outcomes included mortality, morbidity, symptoms recurrence, and endoleak occurrence. Late outcomes included mortality, symptoms recurrence, endoleak occurrence, stent-graft patency, and survival.

Follow-up consisted of clinical examination and duplex ultrasound (DUS) at 1 week; clinical examination and computed tomography angiography (CTA) at 1 and 12 months and yearly thereafter. Median follow-up was 23.77 ± 10 (mean: 22.53; interquartile range [IQR]: 15–28) months.

Statistical analysis. Parametric data are presented as mean and IQR or median and min–max range; absolute values and percentages for nonparametric data. Differences in preoperative and postoperative outcomes were assessed using the Student *t* test. Kaplan–Meier curves were used to estimate survival. Statistical significance was considered at *p* < .05. For Kaplan–Meier curves, standard error exceeding 10% was reported. Statistical analysis was performed using SPSS 16.0 (SPSS Inc., Chicago, IL, USA).

## Results

A total of 21 patients presenting IAAS and managed with the unibody stent-graft system (AFX endovascular AAA system; Endologix Inc.) were included in this study. During the same period, 15 patients presenting IAAS were observed and excluded from the current study. Three of them were managed by conventional open surgery and five by endovascular means without the unibody stent-graft system. Endovascular procedures were performed employing a Cheatham-Platinum (CP) stent (NuMED, Hopkinton, NY) in 2 patients, Viabahns (WL Gore and Associates, Flagstaff, AZ) in 1; and Endurant II stent-grafts (Medtronic Vascular, Santa Rosa, CA) in 2. Four morbid patients were left untreated and managed with the best medical therapy. The remaining three are under surveillance.

The mean age of included patients was 70.62 ± 5 (range 61–82) years, and 19 (91%) were male. A previous heart and/or vascular intervention was reported in 13 (62%) patients. Comorbidities are reported in Table 1. IAAS symptoms were recorded in 14 (67%) patients, including abdominal/back pain in 10 (48%) cases, distal embolization in 6 (29%), and aortic rupture in 3 (14%). Of these symptomatic patients, 1 presented with abdominal/back pain, distal embolization and aortic rupture; 2 with abdominal/back pain and aortic rupture; 1 with abdominal/back pain and distal embolization; 6 with abdominal/back pain; and 4 with distal embolization. In the remaining 7 (33%) cases, an abdominal aorta dilatation >30 mm was the indication to treatment (Table 2).

**Table 1:** Demographics and clinical preoperative data

Number of patients	21
Mean age, years	70,62
Over 70 years, *n* (%)	12 (57)
Female, *n* (%)	2 (9)
Hypertension, *n* (%)	19 (91)
Pulmonary disease, *n* (%)	17 (81)
GOLD 1, *n* (%)	4 (19)
GOLD 2, *n* (%)	4 (19)
GOLD 3, *n* (%)	8 (38)
GOLD 4, *n* (%)	5 (24)
Cardiac disease, *n* (%)	
NYHA I, *n* (%)	6 (29)
NYHA II, *n* (%)	8 (38)
NYHA III, *n* (%)	4 (19)
NYHA IV, *n* (%)	3 (14)
ASA III, *n* (%)	14 (67)
ASA IV, *n* (%)	7 (33)
Renal Function Impairment, *n* (%)	4 (19)
Mean GFR (mL/min/1.73 m^2^)	52
Dialysis	2 (9)
Lipid disorder, *n* (%)	12 (57)
Diabetes mellitus, *n* (%)	6 (29)
Peripheral arterial disease, *n* (%)	15 (71)
Cancer, *n* (%)	7 (33)

*GOLD* Global Initiative for Chronic Obstructive Lung Disease, *NYHA* New York Heart Association, *ASA* American Society of Anaesthesiologists, *GFR* glomerular filtration rate

**Table 2:** IAAS clinical presentation and anatomic features

Number of patients	21
Symptoms, *n* (%)	14 (67)
Abdominal/back pain, *n* (%)	10 (48)
Worse pain ever felt	7
Acute onset	9
Lacerating	6
Migratory	4
Distal embolization, *n* (%)	6 (29)
Aortic rupture, *n* (%)	3 (14)
Asymptomatic, *n* (%)	7 (33)
Aortic dissection, *n* (%)	11 (53)
Intramural hematoma, *n* (%)	3 (14)
Penetrating aortic ulcer, *n* (%)	7 (33)
Associated dilated abdominal aorta (>3 cm), *n* (%)	11 (53)
Iliac artery involvement, *n* (%)	13 (62)

The CTA scan was a diagnostic tool used to confirm the IAAS in all patients and to plan an operative strategy. In patients who had distal embolization, CTA was performed after clinical vascular evaluation and a duplex ultrasound of the lower limbs. For asymptomatic patients, 5 were already inserted into a follow-up protocol; the remaining 2 were incidentally diagnosed.

**Fig. 1:**
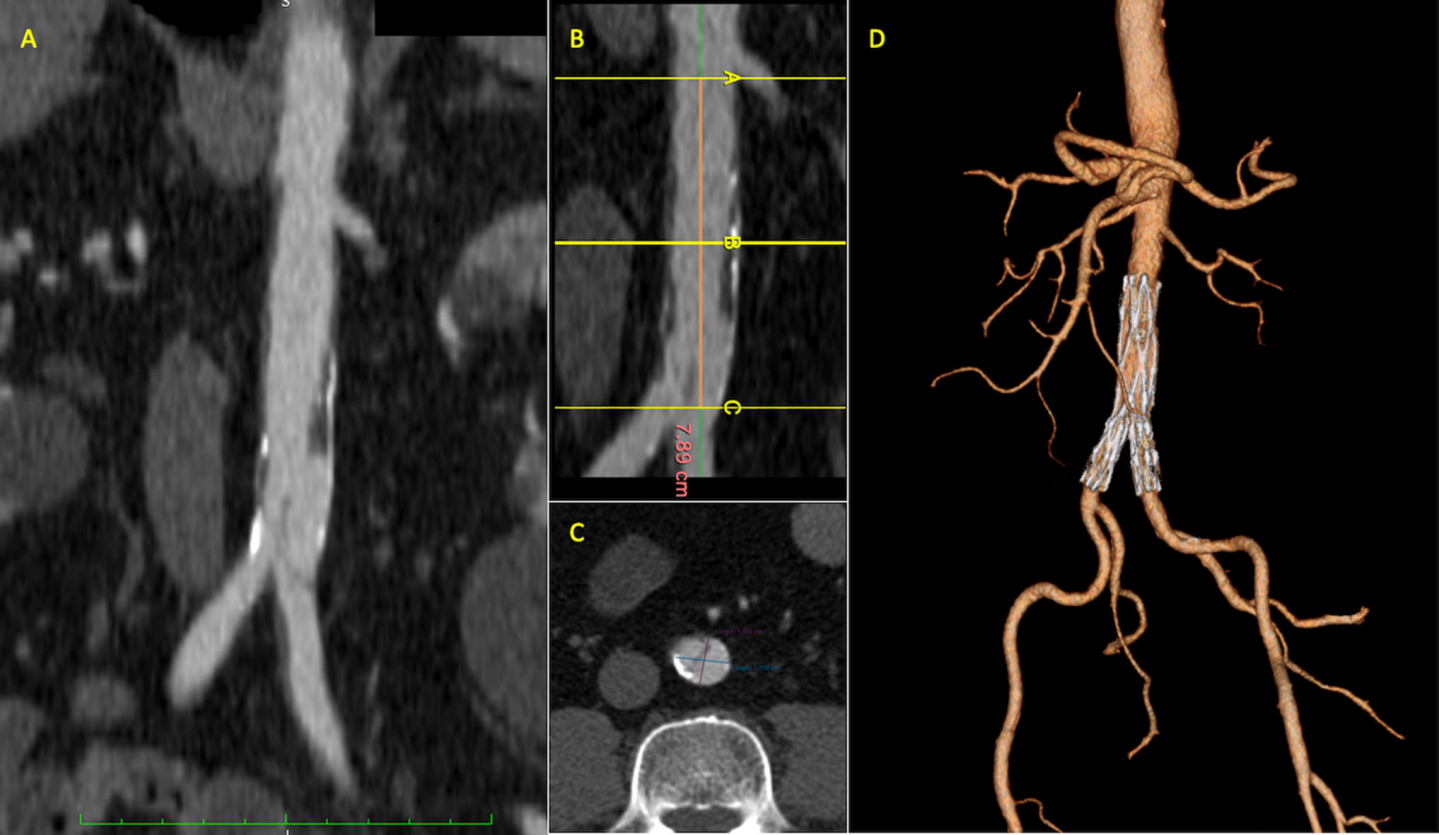
Preoperative CTA with maximum intensity projection (MIP) showing an infrarenal aortic dissection, involving of the origin of the left common iliac artery (**a**); centerline showing a limited length of the infrarenal aorta (less than 8 cm) (**b**); axial projection showing the dissection flap in correspondence of the distal abdominal aorta with limited aortic diameter (approx. 17 × 15 mm) (**c**); postoperative 24-month follow-up CTA 3D reconstruction showing the exclusion of dissection with patent true lumen and disappearance of the false lumen (**d**)

**Fig. 2:**
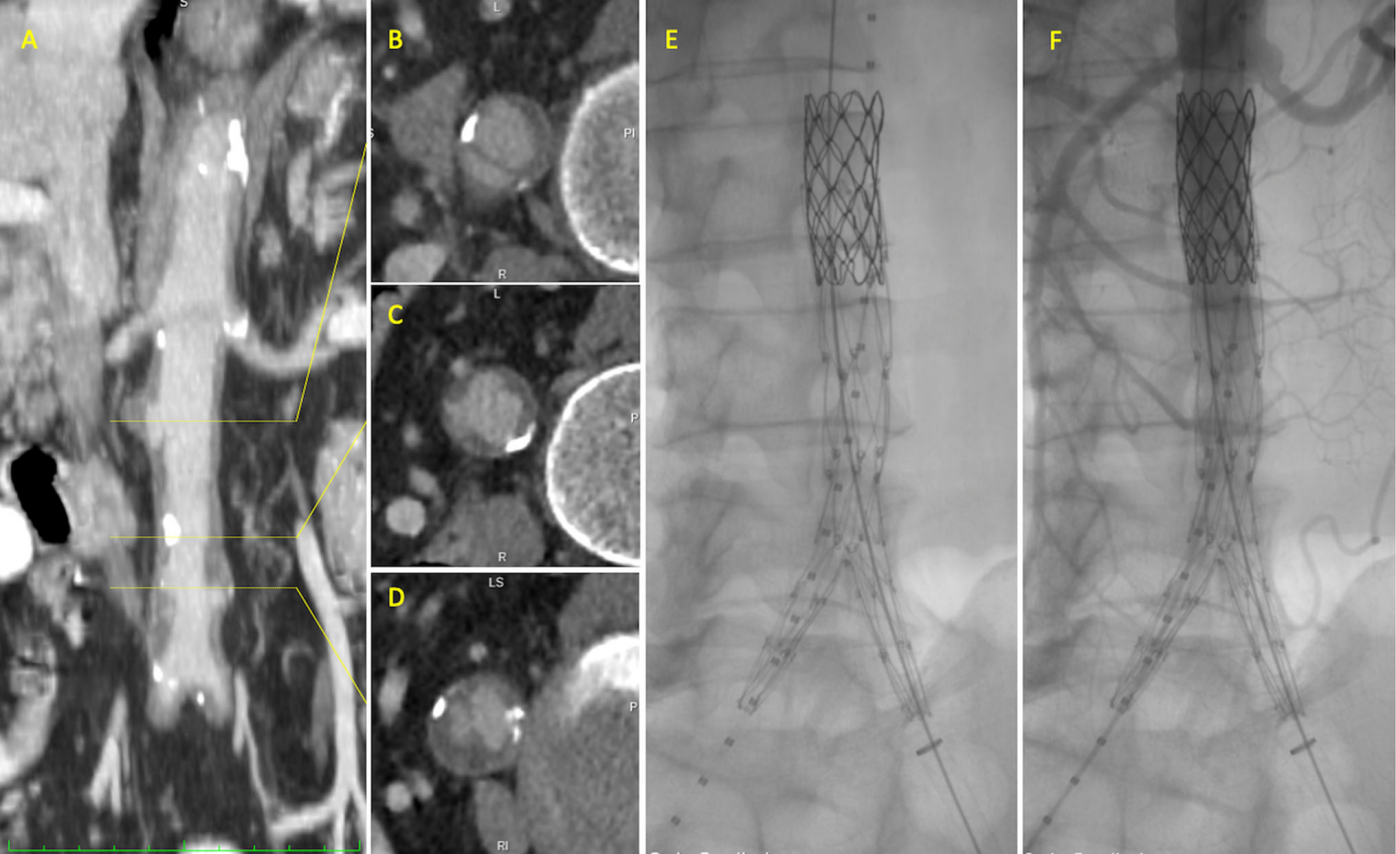
Preoperative CTA MIP showing overall infrarenal acute aortic syndrome (**a**). Infrarenal aortic dissection (**b**), intramural hematoma (**c**) and penetrating aortic ulcer (**d**), intraoperative arteriogram after unibody stent-graft deployment and proximal extension (**e**), and after contrast media injection (**f**)

An AD was recorded in 11 (53%) cases, an IMH in 3 (14%) and a PAU in 7 (33%). An associated dilated (>30 mm) abdominal aorta was detected in 11 (53%) patients (4 symptomatic and 7 asymptomatic); and a dissection of the iliac artery in 13 (62%) (Table 2). In 4 (19%) patients, the distance from the lowest renal artery to the aortic bifurcation was less than 80 mm; in 7 (33%) patients, the mean aortic diameter at iliac bifurcation was less than 16 mm; the combination of both anatomic findings was observed in 4 (19%). The main imaging findings are reported in Table 3. In the present study, the unibody stent-graft system (AFX endovascular AAA system; Endologix Inc.) was introduced and deployed according to the standard technique for abdominal aortic aneurysm repair (Fig. 1). In 13 (62%) cases, a proximal extension was planned. In 9, the proximal extension was undertaken using a VELA infrarenal cuff (Endologix, Inc.); in 2 a covered CP stent (NuMED Inc.); and in the remaining 2 an Endurant II (Medtronic) proximal extension (1 aortic tube and 1 aortic cuff) (Fig. 2). A proximal extension was decided at operator discretion according to the anatomic findings of the proximal neck. Aortic body and proximal extension features are reported in Table 4.

**Table 3:** IAAS imaging findings

	Mean (IQR)
Lowest renal artery to aortic bifurcation distance, mm	88.2 (81–95)
Aortic bifurcation diameter, mm	18.5 (16–20)
Maximal aortic diameter, mm	29.3 (20–42)
Right iliac artery distal landing zone diameter, mm	11.1 (9–13)
Left iliac artery distal landing zone diameter, mm	10.9 (9–12)
Proximal infrarenal neck angulation, °	16.3 (11–23)
Lowest renal artery to primary entry site distance, mm	28.4 (25–33)
Proximal neck diameter al lowest renal artery	20.9 (18–24)

*IQR* interquartile range

**Table 4:** Aortic body and proximal extension features

	Mean (IQR)
Aortic body length, mm	78.6 (70–80)
Aortic body diameter, mm	24.1 (22–25)
Aortic body iliac length, mm	36.3 (30–40)
Aortic body iliac diameter, mm	16.2 (16–16)
Proximal cuff length, mm	76.1 (75–80)
Proximal cuff diameter, mm	27.1 (25–28)

In 10 (48%) patients, a post-dilation of the unibody stent-graft iliac limbs was planned. In 3 patients, the dilation was performed by kissing balloon of the aortic bifurcation.

Interventions were carried under local anesthesia in 19 (90%) patients; general anesthesia in 1 (5%); and spinal anesthesia in 1 (5%). A totally percutaneous approach was employed in 17 (81%) patients according to the standard technique [13]. The mean intervention duration was 108 ± 43 IQR: 75–135) minutes. In the 11 (52%) AD cases, the intravascular ultrasonography (IVUS) was employed.

During the hospital stay, all patients received prophylactic low molecular weight heparin (LMWH) and single antiplatelet therapy (100 mg acetylsalicylic acid). After discharge, the single antiplatelet was continued. For patients reporting preoperative anticoagulation, a switch to therapeutic LMWH before the intervention was performed. The medical management included ß-blockers preferentially administered to control blood pressure and statins for lipid disorder.

**Fig. 3:**
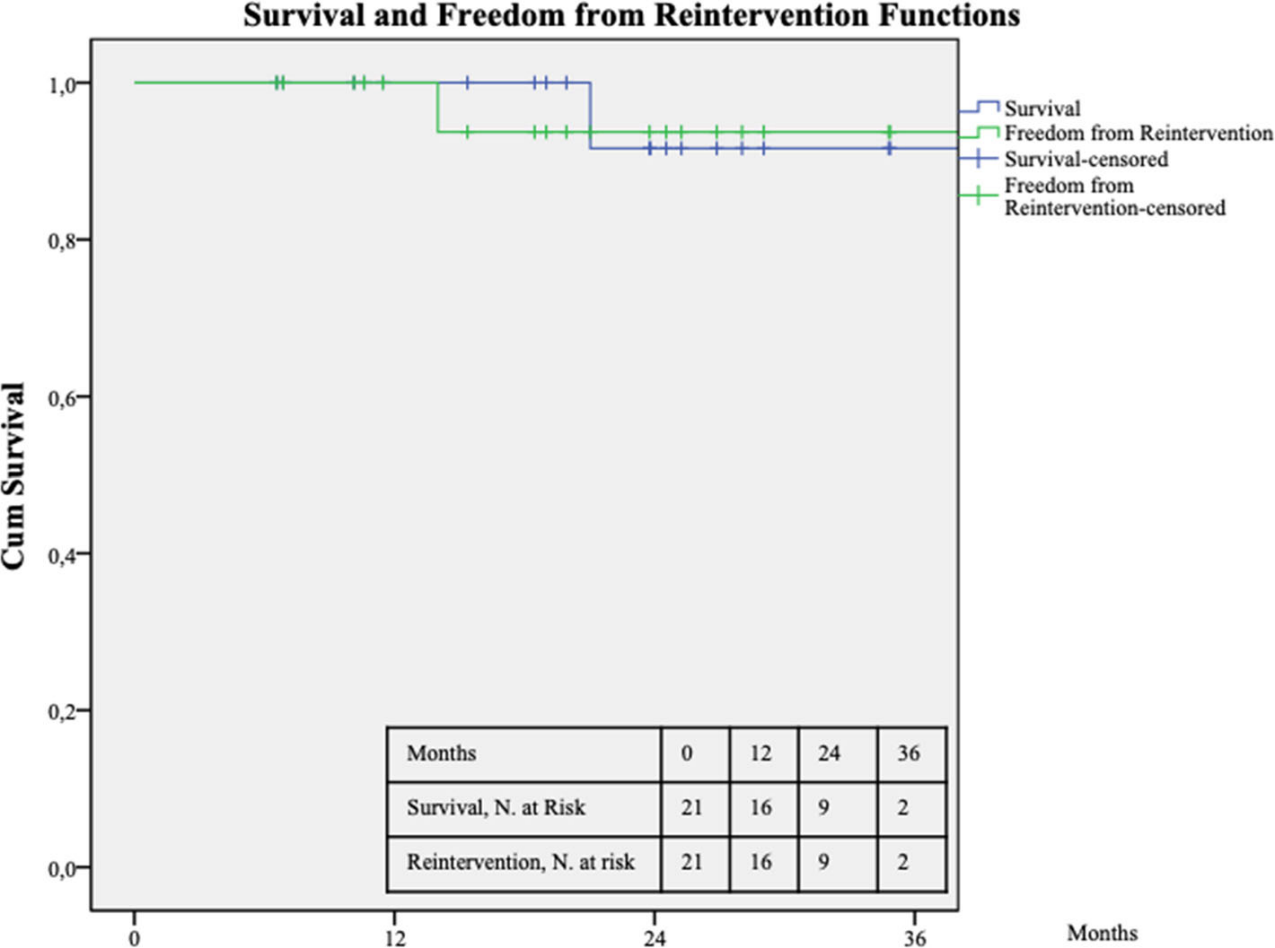
Survival and freedom from reintervention estimated 3-year Kaplan–Meier curves. Standard error does not exceed 10% at 3 years for both survival curves

Technical success was achieved in all cases. No perioperative mortality was registered. In 1 (4.8%) patient, a common femoral artery occlusion requiring a patch angioplasty and distal embolectomy on 2nd POD occurred. No symptomatic recurrence or early endoleaks were detected. During the follow-up, 1 (4.8%) patient died after 21 months of cancer. At 14 months of follow-up, 1 patient required balloon-expandable stent placement for right iliac limb stenosis and claudication. No endoleak or aortic stent-graft occlusion were recorded. At 36 months, the estimated survival and freedom from reintervention were 92% (CI: 37–43; SE: 1.7) and 94% (CI: 37–44; SE: 1.7), respectively (Fig. 3).

## Discussion

AAS is generally reported in the thoracic aorta; among all AAS, the prevalence of classic AD is the highest over IMH and PAU, respectively, with a prevalence of 85–95%, 5–27%, and 2–7% [14, 15].

Current literature lacks incidence data on AAS of the abdominal aortic segment. Most of the publications address IAAD, PAU, or IMHs issues separately and the majority of them are on IAAD [5, 6]. A recent systematic review estimated an IAAD incidence of 5,1 per 1,000,000 persons annually, and an infrarenal aortic segment localization in 82% [5].

Overall, the literature reports up to 500 cases of IAAD and 300 cases of abdominal PAU [5, 6, 16].

Somehow in line with our study, we had an IAAD incidence of 53%, abdominal PAU 33%, and abdominal IMH 14%. From it, a speculative incidence of IAAS could be reported of 10 persons per 1,000,000 annually.

Anyway, IAAS is usually reported in males over 60 years and can be secondary to trauma, iatrogenic, or spontaneous [17]. The natural history of IAAS is a rupture in up to 15% of patients and a mortality rate ranging from 17 to 28% [18].

As recommended by Farber et al., a 3-cm abdominal aortic diameter should be considered for elective treatment due to the higher rupture risk compared to abdominal aortic aneurysm (AAA) and the unknown natural history of aneurysmal degeneration of the dissected abdominal aorta [19].

IAAS can be managed with the medical, surgical, or endovascular approach. An insight from the IRAD reports that the medical management on 18 patients presenting an IAAS was significantly associated with a higher mortality risk when compared to operative treatments, suggesting an aggressive surgical or endovascular repair [9].

The endovascular approach showed better results with a reduced mortality and morbidity risk when compared to conventional open surgery [5, 6, 17].

The main concerns of IAAS endovascular treatments are the lack of dedicated devices and the use of standard aortic stent-grafts outside the instruction for use (IFU). Different solutions including uncovered stents, covered stents, and stent-grafts have been proposed [18].

Due to the risk of distal embolization, the use of uncovered stents is limited to patients presenting IAAS without thrombus. Covered stents and stent-grafts are employed in most patients; moreover, the use of stent-graft is mandatory in case of associated infrarenal aortic aneurysm [20].

IAAS management with different types of aortic stent-graft has been associated with good results, even in terms of aortic remodeling and false lumen thrombosis; however, limb occlusions and persistent endoleaks are the main cause of reinterventions [5, 6, 21].

IAAS generally presents specific anatomic features including a short/limited length of the infrarenal aorta, and a narrow arterial lumen, especially at aortic bifurcation and iliac arteries. The association with aneurysmal dilation generally worse the IAAS anatomy; in these circumstances, the aortic true lumen is further narrowed by the false lumen from which the aneurysmal dilatation generally arises [22].

To overcome these anatomic limitations, different endovascular solutions including the use of aortic tube, aortic kissing stent-grafting, or standard aortic stent-graft have been reported. The use of aortic tube can be indicated in IAAS with no iliac involvement with a potential risk of migration due to the lack of distal iliac fixation. The limitations of the kissing stent-graft technique are related to the shape changes of the abdominal aorta, the risk of proximal misaligning, and the risk of occlusion.

The usual IAAS anatomic features represent major limitations also to the available standard bi/trimodular aortic stent-grafts. In particular, the short distance from the lowest renal artery and the aortic bifurcation has the risk of contralateral gate opening inside the homolateral iliac introduction site; the narrowed diameter in correspondence of the aortic bifurcation has the risk of contralateral gate incomplete opening or collapse inside the distal aorta. Aorto-uni-iliac stent-grafts represent another option but require an additional femoro-femoral bypass.

A recent publication reported IAAD treatment with Aegis™-B unibody stent-graft (Microport, Shanghai, China) in 32 patients, with good results, no events of mortality within 90 days and 1 limb occlusion [23]. Moreover, they reported the use of proximal cuff extension only in 3/32 (9.3%) case, in contrast with our report of 13/21 (62%), most probably due to the shorter aortic length of Asiatic population. However, Aegis™-B (Microport) is not available on the EU and US markets.

In our experience, the best commercially available device adapting to IAAS characteristic was the unibody stent-graft system (AFX endovascular AAA system; Endologix Inc.). The bottom-up construction of such a device allows its use in small aortic bifurcation diameter and short renal to aortic bifurcation distance. Further, the unibody stent-graft system (AFX endovascular AAA system; Endologix Inc.) avoid the gate cannulation issues related to conventional bi/trimodular stent-grafts in such a small aortic diameter (mean 29.3 mm) as reported in our cohort.

These anatomic issues are uncommon for AAA intended for treatment (>5–5.5 cm), exploiting greater infrarenal aortic length and intra-aortic space for device maneuvers. In fact, the AFX endovascular AAA system (Endologix Inc.) device is designed to treat AAAs and IFU does not include IAAS. The recent FDA concerns about type III endoleaks have prompted the company to review and issue security updates for AFX (Endologix Inc.) devices. The investigations into type III endoleaks identified several contributing factors, including an inadequate component overlap and a large or tortuous aorta: on these bases, Endologix introduced an IFU update on sizing algorithm for the AFX (Endologix Inc.) platform to maximize overlap, calculated on the aneurysm radius +20 mm.

Obviously, the stated indications regarding the endoleak issues are irrelevant in the case of IAAS treatment. Theoretically, IAAS follow-up should be uneventful for type III endoleaks and further investigation in regard could clarify the role of the AFX endovascular AAA system (Endologix Inc.) device.

In this series, 15 (71%) patients exhibited at least one anatomic feature not fitting for conventional bi/trimodular bifurcated graft. Based on this experience, the IAAS anatomic limitations to the use of the unibody stent-graft system (AFX; Endologix Inc.) are principally related to the distance from the aortic bifurcation to the origin of the internal iliac arteries (IIAs), as *a* < 30 mm length pones the risk of IIA occlusion. Restricted aortic bifurcations (<14 mm) also represent an increased risk of guiding each limb into its respective common iliac artery.

IVUS is an additional endovascular tool to identify the entry tear of AD, vessel diameters and lengths [24, 25]. In our practice, this device was useful to confirm guidewire intraluminal route and it was employed in all patients (52%) presenting with IAAD.

Limitations of the present study are the retrospective analysis and the limited number of included patients. The major limitation is the absence of a comparative groups treated by open surgery or other endovascular means. In addition, a relevant bias is the lack of randomization of the approach chosen, including open surgery/unibody stent-graft/trimodular stent-graft/covered stent-grafts. Also, the follow-up duration is a limitation to drawing robust conclusions.

## Conclusions

In the reported experience, the endovascular treatment of IAAS was safe and effective with promising mid-term outcomes. The use of unibody stent-grafts expands the endovascular indication despite the usual anatomic IAAS features. Larger studies with longer follow-up are required to validate the outcomes of the reported technique.
